# Falls from a balcony while intoxicated: a new injury trend among young adults?

**DOI:** 10.1186/s40621-019-0181-3

**Published:** 2019-02-11

**Authors:** Kathryn B. Schaffer, Gary Schwendig, Fady Nasrallah, Jiayan Wang, Jess F. Kraus

**Affiliations:** 1 Scripps Memorial Hospital La Jolla, 9888 Genesee Ave., LJ601, La Jolla, CA USA; 2 Department of Epidemiology, UCLA, Los Angeles, CA USA

**Keywords:** Balcony falls, Fall from height, Trauma, Alcohol

## Abstract

**Background:**

Unintentional falls from heights, including balconies, result in life threatening traumatic injury. Alcohol, when combined with environmental factors and poor judgement, can potentially lead to fatal outcomes. One trauma center’s registry identified a group of young adults falling from balconies and we investigated the role of alcohol.

**Methods:**

Hospital trauma service admissions from 2010 through 2017 were reviewed for unintentional falls from heights. Suicide attempts and unintentional falls off ladders or roofs were excluded. Data were obtained from trauma registry and medical record review, as well as social work service interviews.

**Results:**

Falls from heights comprised 4.8% of injuries treated at our trauma center during the eight-year study period with 98.5% admitted. Of patients admitted because of falls, 10.3% (55/532) were from a balcony. The majority of this group of patients was male and 19–29 years old (67%). Of patients with a blood alcohol concentration (BAC) determination, 62% had a positive BAC upon hospital admission with an average of 0.20 g/dL among those 34 patients. No gender differences were evident for alcohol use. Seven of the eight patients under the legal drinking age of 21 years were a subgroup with high alcohol use as compared with patients 21 years and older (*p* = 0.099). Ninety-four percent of falls occurred at residential locations such as dormitories or apartment complexes, often during a social event. Backward falls off railings and attempts to jump to adjoining balconies were common. Head, thorax/abdomen, and extremity fractures were common, with an average injury severity score (ISS) of 16. Average length of hospital stay was 8 days. Most patients (67%) were discharged home after hospital stay, but 21% were transferred to inpatient rehabilitation or skilled nursing facilities. There were two deaths.

**Conclusions:**

Falls from balconies among young adults occur in our area yet the true frequency of these events remain unknown. Occurrence was most common among underage drinkers. Generalization is difficult with this small sample, yet high risk behaviors and environmental factors were evident. It is imperative that educational programs focus on this population with collaborative prevention efforts focused on the dangers of, and increased risk of injury associated with the balcony environment.

## Background

Globally, an estimated 37 million unintentional falls requiring medical treatment occur each year (World Health Organization 2018). Falls are third leading cause of injury-related deaths in the United States and they are a growing public health problem among intoxicated young and middle-aged adults (Centers for Disease Control and Prevention 2016a, b).

Alcohol is a major risk factor for fall-related injuries. More than one-half of non-occupational falls in adults are associated with alcohol use (Mosenthal et al. 1995). Consumption of alcohol is an actuating factor and is estimated to double the severity of traumatic brain injury (TBI) (Kumar and Srivastava 2013; Moore 2005). The United States Department of Agriculture’s Nutrition Evidence Library showed that there is strong evidence that drinking increases the risk of unintentional falls, motor vehicle crashes, and drowning (What is the relationship between alcohol intake and unintentional injury? 2016). Research has shown that alcohol and drug intoxication increase the risk of falls and risk of severe craniofacial and limb injury (Johnston and McGovern 2004; Kool et al. 2009; Hingson and Howland 1987). In addition, underage drinking may impair neurological development, causing people to make irresponsible decisions, have memory lapses, or process neural impulses more slowly (Crowe et al. 2012). Underage drinking is common, and severe injury related to its use is always a concern that warrants attention.

Among 18–24 year olds in the United States, it has been estimated that over 63% of unintentional injury deaths are alcohol-related falls (Hingson et al. 2009). This estimate includes falls from any height. The occurrence of intoxicated students who fall from high places, such as dormitory or apartment balconies, and are injured or killed while at parties has increased over the years (Peterson 2016; Canales 2015; Two college students charged with alcohol violations in fatal fall of a freshman who slipped from tenth-floor balcony to her death after attending ‘all-you-can-drink’ fraternity party 2014; Falls From Fraternity Houses Turn Out To Be A Big Problem On College Campuses 2014; Walters 2012; Pateras 2016; Sokol 2016). One recent study in the United States found that an estimated 5600 balcony-related falls involved structural failure of the balcony (Shields et al. 2011).

Although previous studies have addressed alcohol-related falls, none have addressed unintentional alcohol-related falls from balconies among young and middle-aged adults. Information on this question could help identify appropriate interventions. Our experience at one Level II trauma center in San Diego, California identified a group of young and middle-aged adults who fell from balconies and suffered severe injury. The aims of this exploratory study were to describe the common factors associated with these falls, to identify the behavioral factors involved and describe injury outcomes.

## Methods

Our hospital is an ACS (American College of Surgeons) verified Level II Trauma Center in San Diego with an exclusive trauma patient catchment area of 479 mile^2^ located to the north-west of downtown San Diego, California. All trauma admissions, regardless of age, from January 1, 2010 through December 31, 2017, were reviewed to identify patients. Data on patient injuries from falls prior to 2010 were not included in this study base because of incomplete information.

External Cause of Injury Codes (ICD-9 and ICD-10) for falls from a height and from a building or structure were selected. Injury descriptions including details within the text of a balcony-related fall were carefully reviewed and coded. In as much as official reports of falls were routinely not undertaken we sought information on heights of balconies from university architecture or maintenance personnel. The heights of the falls were coded as less than or greater than twenty feet. Balconies located on the first story were conservatively estimated to be under twenty feet high, and all others were coded as greater than twenty feet. All intentional falls from suicidal behavior, or falls from ladders, scaffolding, through roofs or windows were excluded.

Medical record and trauma registry data were reviewed and abstracted for aspects of prehospital care, injury body location, demographics, diagnoses, procedures, injury severity score (ISS), blood alcohol concentration (BAC), length of stay (LOS), and hospital charges for care. Use of alcohol was determined in three ways. First, a positive BAC, second, written notations in pre-hospital or the hospital medical record, and third, notes by the social worker during the patient interview. Blood alcohol concentration was the only drug assessed in routine emergency department analyses.

Data are presented as the mean + standard deviation (SD) or unadjusted percentages, where appropriate. Although age, ISS, LOS, and distance in falls and BAC were reported or observed in continuous numbers, the small sample size dictated the use of categorical data in this exploratory analysis. Categorical data were analyzed using a two-sided Fisher exact test. All *p*-values < 0.05 were considered statistically significant. This study was approved by the study hospital’s Institutional Review Board.

## Results

**Fig. 1:**
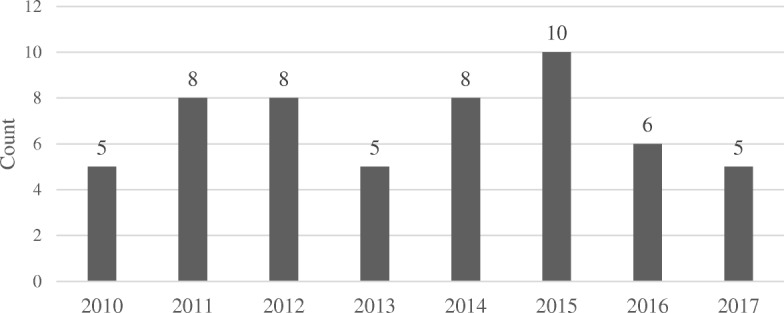
Incidence of falls from balcony by year, *N* = 55

Over 11,000 patients were admitted during the study period for treatment of traumatic injuries. About one-third (36.2%) of these patients were treated for falls of any type and 13.2% (532) were unintentional falls from a height (Fig. 1).

Balcony-related falls comprised 10.3% (55) of the falls from heights and is the subject of the remaining analyses. Two-thirds of patients were males and 18 to 29-year olds were 58% of the study group.

Ninety-four percent of falls occurred at a residence. These residences include college campuses, military base apartment housing and private multi-story apartments and homes. Falls from a balcony in our patient group ranged from single to nine stories. The majority of falls (65%) were from a second story or higher balcony and 35% were falls from one story. Activities prior to injury included falling backwards off railings (while sitting facing the residence) or attempts to “jump” from one balcony to another or jumping to adjacent trees. Four patients fell due to failure of the balcony railing while they were sitting on or leaning up against it. Fifty-eight percent of the events (32) were unwitnessed falls, yet the patients were not necessarily alone at the time (Table 1).

**Table 1:** Study cohort and fall event characteristics

	Number
Age	
18–29 years	32
30 years and older	23
Gender	
Female	18
Male	37
Injury location	
Residence	52
Non-residence	3
Fall event occurrence - day of week	
Thursday - Sunday	34
Monday - Wednesday	21
Fall event witnessed vs unwitnessed	
unwitnessed fall	32
witnessed fall	23

Many of these events occurred during a social event with many guests inside the residence, unaware of a fall from the balcony. Average time from the fall to arrival at the hospital was 1.75 h, and ranged from 18 min to 17.5 h. Majority of patients (67%) arrived to the hospital Emergency Department (ED) within 1 h of the fall, and 23% arrived between one and 4 h. Nine percent of the patients arrived at the hospital more than 5 h after the fall. In these cases, peers found the patient after the fall and brought the patient back to the residence before seeking medical assistance. The majority of the all falls (63%) occurred between 9 PM and 4 AM.

All of the falls in the study group involved alcohol use in some way, either by documentation in pre-hospital or in-hospital records or alcohol being consumed at the location at time of the fall or a positive BAC documented in the medical chart. Of those with a BAC determination, 34 (62%) of patients had a positive BAC upon hospital admission with an average of 0.20 g/dL. No gender differences were evident in alcohol use, ISS or hospital length of stay in our patient group. Seven of the eight patients under the legal drinking age of 21 years had a positive BAC (*p* = 0.099) (Table 2).

**Table 2:** Positive BAC, injury severity and length of stay by gender and age

	+ BAC (N)	Mean BAC (g/dL)	Mean ISS + SD	Mean LOS (days)
Female *n* = 18	9	0.23 SD 7.25	14.9 SD 8.43	5.7 SD 5.51
Male *n* = 37	25	0.20 SD 0.106	17.2 SD 16.2	9.9 SD 15.0
18–20 years	7	0.23 SD 0.114	15.6 SD 8.50	8.6 SD 10.5
21 years and older	27	0.20 SD 9.497	15.0 SD 11.1	7.7 SD 14.7

Most injuries were to the head, thorax/abdomen, and extremities with a mean ISS of 16 and mean LOS of 8 days. Head injuries occurred in 64% of the patients and ranged from concussions to severe brain injury such as subdural hemorrhages, cerebral contusions, and skull fractures. Twenty-three patients (41%) required surgery for orthopedic injuries and/or brain trauma. Thirty-eight percent of all patients required intubation during their hospital stay.

Most patients (67%) were discharged to their residence after their hospital stay but 21% were admitted to an inpatient rehabilitation facility or skilled nursing facility. There were two in-hospital deaths. Total charges for initial hospital stay ranged from $11,293 – $1.8 million, with an average charge of $280,480.00 per patient. Data were not available for additional medical expenses after discharge, including rehabilitation services.

## Discussion

Unintentional falls from balconies are a distinctive type of fall but not uncommon as previously thought. A closer look at the behaviors and demographics of the injured population shows the commonalities. In addition to falling off of balconies while under the influence of alcohol, victims are seldom alone prior to falling and often attended social events with peers. Because the fall event is sudden during these social events where others are also drinking and distracted with the party atmosphere, these occurrences are seldom witnessed and, in many cases, result in a delay to medical treatment.

A similar public health issue called “balconing” has been reported from some European vacation locations (Segura-Sampedro et al. 2017; Pérez-Bovet et al. 2015). This term refers to the practice of tourists jumping from hotel balconies or roofs into swimming pools, or between hotel balconies (BBC Newsbeat 2016). Although “balconing” specifically relates to a playful situation during a tourist holiday, it has similarly caused severe trauma in young groups of people, especially where alcohol intake, is apparent. Postings in social media of “balconing” have increased and have spread the popularity of this very dangerous behavior. In the United States, “balconing” has not been recognized as a problem compared with Europe. However, unintentional falls from balconies among young adults, on or near college campuses, or while participating in a college-related event has been recognized.

Search in the media and internet produced a large number of incidents throughout the United States and often involved young inebriated adults on, near, or in a college campus-related social activity. One example was a patient who fell from a third-story dormitory balcony after drinking excessively at the university near our trauma center. The patient’s injuries were very serious, and included a fractured skull, extensive chest trauma, and multiple extremity fractures. The patient had an extensive hospital stay and rehabilitation, and eventually returned to school. The event was life changing. Grateful for survival and recovery, the patient shared the experience on social media in a personal outreach to peers about the dangers of underage drinking. Social media has been used to spread the news of these types of events, especially among young adults. More common than an educational posting about dangers of alcohol and risky behavior are postings of dangerous activities, often involving alcohol and drugs. Unfortunately many of these postings of high risk behavior have led to copy-cat actions to mimic the behavior.

As this study has summarized, balcony-related falls that have resulted in severe injuries have costly outcomes, not only in monetary terms, but include lost time from school and work. There is, of course, loss of a productive life for those who die or become disabled due to their fall injuries. Although the number of patients injured from falls from balconies is small in this report compared with other fall-related mechanisms, these events continue to occur with a risk of a fatal outcome. Balcony falls are a preventable public health problem that should not be ignored.

Universities generally have strict policies for students, under 21 years of age, on the possession, purchase, and consumption of alcohol and being intoxicated while on campus. Although these policies are directed toward students who reside on campus, parties involving alcohol still occur and often in campus housing. Multi-story dormitories and university affiliated apartments on or near universities are common and, as universities continue to expand and build more multi-level housing options, more balconies and risks are available. While most educational institutions provide structured alcohol and drug prevention programs for students, especially those living on campus, the issue of drinking in residences and environmental risks related to balconies needs to be an active part of the curriculum.

As shown from the collected social worker interviews with patients, peers present at time of the fall event frequently demonstrate poor decision-making with respect to assisting the injured person. That is, they transport the injured back to the resident dormitory without a report to emergency responders. The reasoning for this is unclear, but interviews conducted by hospital staff with the patient often described that all members of the “party” might have been intoxicated and feared disciplinary action for underage drinking. Unfortunately, the decision to not report these injuries leads to delay in medical treatment and likely under reporting of the true number of cases with the potential exacerbation of the injuries themselves.

The university near our trauma center, where several patients have fallen from a dormitory or apartment balcony on campus, has begun to address this problem of balcony falls as part of their residential life program. Information is provided to students at time of orientation to increase awareness about risks associated with balconies (and drinking) in their housing. Information is also directed at the limits of the number of people on a balcony at one time, the hazard of sitting on a rail, and climbing or jumping from balcony to balcony. Rules on drugs and underage alcohol consumption are addressed as part of normal college student orientation, but with the increase in balcony falls on campus, the combined hazard of a balcony environment and alcohol consumption are now part of residential life orientation. This education also includes the critical importance and responsibility of students to assist and alert authorities without delay when medical attention is warranted rather than avoiding or reporting the incident for fear of the consequences. Another means of outreach for students on campus in the past few years was sporadic social media alerts about the hazards of drinking and awareness of the party environments, including “hanging out” on the balcony.

## Limitations

Generalization of our findings was difficult because the small sample size and restriction to one community as represented in our hospital data source. The actual frequency of these events may be underreported in our community and possibly nationwide based on unsolicited comments from colleagues who offered their own observations at recent conferences where this subject was presented. The effort to identify falls from a balcony was challenging in our trauma database. There was often no pre-hospital (event) information available since many patients were transported to the hospital by private vehicle. There was a delay in medical treatment for a third of the cases, a disturbing statistic given the severity of injuries involved. Reconstructing the story of what happened at time of injury was dependent on the availability of pre-hospital documentation (if pre-hospital treatment was provided), social worker interviews with the patient, and any documentation by the physician or other medical staff. We found that external mechanism of injury coding for ICD-9 was often miscoded for any type of fall prior to 2017. Thus study years, 2010–2016, utilizing ICD-9 coding required additional searches to confirm mechanism and place of injury. ICD-10 external cause of injuries are more detailed and list balcony as an option, “W13.0XXA - Fall from, out of or through balcony”. Although mechanism of injury was often correctly coded using ICD-10 coding, there was still some minor misclassification of balcony falls, but much less as compared to ICD-9 where balcony related falls were often coded as E882, “Fall from, out of or through a building”.

The exact number of deaths at the scene of the fall are unknown although a thorough search of written media for deaths related to balcony-falls during the time period was undertaken. The San Diego County Medical Examiner summarized death rates from unintentional falls from 2010 to 2016, grouping all falls together regardless of height. If any age group had less than five fatalities per year, death rates are not calculated. From 2010 to 2016, there were no death rates provided for age groups less than 65 years of age. Thus the report acknowledges five or less unintentional fall related deaths per year for decedents under 65 years of age (Wagner 2016). Presenting a fall-related death rate among our cohort was not possible and is another limitation of this study. As a result of our investigation of all fall from heights, we now communicate directly with the County Medical Examiner on these and related types of deaths.

## Conclusions

Without intervention, fall-related injuries and deaths from balconies will no doubt continue. It is imperative that injury prevention programs address this type behavior regardless of age. The safety of the balcony in light of previous recorded falls must be reassessed. The type and heights of railings should be reexamined. Future work will incorporate national and state data, as well as our County registry for development of collaborative prevention efforts that involve our local universities as well as privately owned multi-level residences.

## Abbreviations

BAC: Blood alcohol concentration
ED: Emergency Department
ICD-10: International Classification of Diseases, 10th Revision
ICD-9: International Classification of Diseases, 9th Revision
ICU: Intensive care unit
ISS: Injury severity score standardization of severity of traumatic injury
LOS: Length of stay in day units
SD: Standard deviation
